# Genetic Characterization of Wild Soybean Collected from Zhejiang Province in China

**DOI:** 10.3390/genes16070776

**Published:** 2025-06-30

**Authors:** Xiaomin Yu, Xujun Fu, Qinghua Yang, Hangxia Jin, Longming Zhu

**Affiliations:** 1 Institute of Crop and Nuclear Technology Utilization, Zhejiang Academy of Agricultural Sciences, Hangzhou 310021, China; fuxj@zaas.ac.cn (X.F.); yangqf@zaas.ac.cn (Q.Y.); jinhx@zaas.ac.cn (H.J.); zlmsllzly@163.com (L.Z.); 2 Hangzhou National Soybean Improvement Center, Hangzhou 310021, China

**Keywords:** wild soybean, origin, germplasm resource, genetic diversity

## Abstract

**Background/Objectives**: Wild soybean could grow in different soil types and under diverse climate conditions, which provides rich genetic resources in the locality. It is important to understand the genetic diversity as well as phenotypic variation for soybean breeding. The objective of this study was to analyze the genetic and phenotypic characteristics of 96 wild soybean accessions collected in Zhejiang Province, and to explore the potential advantage of germplasm resources for further application. **Methods**: These 96 annual type soybean resources have been propagated, identified and evaluated in both 2022 and 2023. In addition, their agronomic, quality and genetic traits have been characterized. **Results**: Most of the accessions exhibited sooty seed coats with different stem and leaf shapes. The means of seed protein and oil contents were 45.4% and 10.0%, respectively. There were significant differences in both protein and oil contents based upon the seed size. The average number of alleles per loci was 3.96, and the average PIC value was 0.619. The 96 accessions were clustered into three different groups. Each group had a consistency with both the geographical sources and the seed quality traits. **Conclusions**: There were agronomic, quality and genetic variations of these wild soybean accessions by the comprehensive analyses. This study gave us a combined understanding of both phenotypic variation and genetic diversity of wild soybean accessions in Zhejiang. Therefore, both reasonable exchanging and crossing between different soybean types is highly recommended.

## 1. Introduction

Cultivated soybean (Glycine max) is an essential crop used as oil, food and forage and it has been domesticated from wild soybean (Glycine soja) in China for over 5000 years. Wild soybean is mainly distributed through China, Japan, Korea and Russia and it is centered in China with a wide geographical distribution [1,2,3]. In China, the vertical distribution is from the West (longitude: 97°) to the East (longitude: 135°) and the horizontal distribution is from the North (latitude: 52°) to the South (latitude: 24°) [4,5]. Therefore, the wild soybean species could grow in different soil types and under diverse climate conditions. More than 8500 wild soybean germplasm resources have been conserved in the National Crop Germplasm Resource Bank of China (Beijing, China) so far [6]. Compared with cultivated soybeans, wild soybeans have their specific characteristics, such as abundant nutrients, strong resistance to stress and multiple flowers and pods. Moreover, both wild soybean and cultivated soybean can be compatible for hybridization [3,4,5]. Hence, wild soybean provides an important gene source for improving quality traits and breeding new varieties of cultivated soybeans. It is also a valuable genetic resource to study the origin, evolution and classification of soybean.

The evaluation of germplasm resources for crops is a frequent research topic all over the world. It is a basic requirement for resource research to meet both breeding and production needs based upon the development and change of agricultural production [6,7]. An et al. [8] studied the genetic variations of a total of 50 accessions of annual wild soybean and cultivated soybean by three genetic fingerprinting systems. Genetic diversity calculation suggested that the diversity of wild soybeans was higher than that of cultivated soybeans. Liu et al. [5] investigated the genetic basis and population divergence of the local adaptation of 72 wild soybean accessions in China. The analysis showed that the population size of Southern China expanded before that of Northeastern and Central China. Sun et al. [9] analyzed allelic profiles of eight core wild soybean accessions from Northern China and 141 annual wild soybean accessions from Southern China. The results suggested that the wild soybean from Fujian province appeared in different groups, and moreover, Fujian province could be the major center of genetic diversity for annual wild soybean in Southern China.

Zhejiang Province is located on the southeast coast of China and next to both Fujian and Jiangxi provinces, with different kinds of landforms, climates and crops. There is a significant role of grain and oil crops in dryland for agricultural production in the province, among which soybean has one of the largest growing areas [6,10]. Soybean has been cultivated for a long history with different types, including spring, summer and autumn types, according to the sowing season [11,12]. Meanwhile, wild soybean is widely distributed with various types all over the province. The collection and investigation of wild soybean resources has started since 1979, and there were significant variations in both leaves and seeds among the materials collected. Although the genetic and phenotypic diversity of cultivated soybean has occasionally been reported, the genetic characterization has seldom been studied and reported in Zhejiang [6].

The wild soybean contains rich genetic resources, which could be a new basis for both development and promotion of soybean breeding in the locality. Therefore, it is an area of current concern about how to transform this potential advantage into a practical advantage [10]. In Zhejiang Province, a total of about 96 wild soybean accessions (annual type) have been collected in recent years. Compared with cultivated soybean, these wild accessions may exhibit greater genetic diversity due to their adaptation to heterogeneous environments and specific quality traits. It is invaluable to understand the genetic diversity for efficient protection and utilization of the germplasm collection. This study focuses on these 96 newly collected wild soybean resources with different seed sizes and weights. They have been propagated, identified and evaluated from 2022 to 2023. Their agronomic, quality and genetic variations have been comprehensively analyzed. This could provide a scientific foundation for the further protection and utilization of wild soybean germplasm resources in Zhejiang Province of Southern China.

## 2. Materials and Methods

### 2.1. Plant Materials

A group of 96 wild soybean accessions (annual species) collected from Zhejiang Province recently were selected to represent the wild soybean genetic stocks in Southeastern China [3,5,10]. The materials were grown in the experimental field of Zhejiang Academy of Agricultural Sciences (Hangzhou, China). They were planted in the late May of 2022 and 2023, respectively. A randomized complete block design was applied with three replicates. For each replicate, plants were grown in two rows with a 1-m row space. Each row was 10 m long with a rate of 4 plants grown per meter. The field management was performed based upon the local habit. The leaf samples were collected two months after seedling emergence. The seeds were harvested at full natural maturity and then air dried for storage for each year [6].

### 2.2. SSR Analysis

Genomic DNA was isolated from the young leaves of each accession using the CTAB method [6,11]. The simple sequence repeat (SSR) analysis was performed based upon the previous studies [7,13]. According to these corresponding reports, the genotyping was conducted using 50 SSR markers. These markers were evenly distributed on 20 soybean genetic linkage groups with 2–4 markers in each chromosome [13]. According to the absence or presence of alleles for each accession, a zero-one data matrix was then created. A distance tree was subsequently constructed based upon the unweighted pair group method using arithmetic mean (UPGMA) cluster analysis [6,11,12].

### 2.3. Trait Analysis

According to the seeds harvested each year (2022 and 2023), plant traits were recorded for each accession, including main stem, pubescence color, flower color and leaf shape. Seed characteristics were also recorded, including seed coat color, seed sootiness and 100-seed weight. The main stem of wild soybean was recorded as apparent or unapparent. The seed coat color was recorded as black, brown or di-color. The leaf shape was recorded as round-ovate or long-ovate. The seed sootiness (bloom) was recorded as absent or present. The 100-seed weight was also calculated in triplicate and then averaged [6,12].

### 2.4. Seed Quality Analysis

For each accession, seed samples (about 100 g) were analyzed by near-infrared reflectance (NIR) spectroscopy. The value of each sample used for further analysis was an average of ten sub-samples measured by NIR using an Infratec 1241 Grain Analyzer (Foss, Sweden). The protein and oil contents were presented as grams per 100 g (%) of dry seed matter unit. The statistical analyses, including correlation and cluster analyses, were conducted using SPSS statistics 19.0 (IBM, Armonk, New York, NY, USA) [6,11,12].

## 3. Results

### 3.1. Seed Characteristics and Quality Traits

To identify the differences in seed characteristics among these 96 accessions, seven corresponding traits were investigated, including main stem, leaf shape, flower color, pubescence color, seed coat color, seed sootiness and 100-seed weight. The phenotype of the stem was twining for wild soybeans, and 61.5% of the accessions had an obvious main stem. The percentages of round-ovate and long-ovate leaves were 47.9% and 52.1%, respectively. There was no variation for either pubescence color or flower color, and all accessions exhibited both purple flowers and brown pubescence. The sootiness of the seed coat is a specific character for wild soybeans. The percentages of sooty and non-sooty seed coats were 85.4% and 14.6%, respectively. Therefore, the agronomic diversity of wild soybean was not comparable to that of cultivated soybeans.

**Figure 1:**
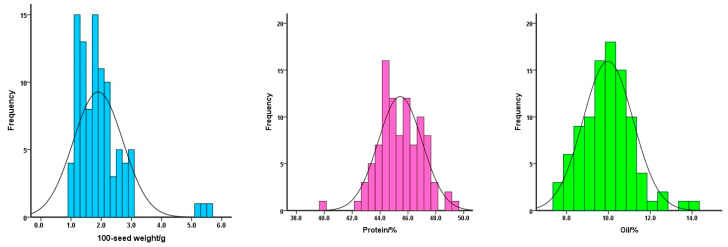
Distributions of 100-seed weight (**left**), protein content (**middle**) and oil content (**right**) of the total wild soybean accessions.

To gain insight into the quality profiles in soybean seeds, we analyzed 100-seed weight, protein content and oil content in seeds of these 96 accessions (Figure 1). The 100-seed weight ranged from 1.0 to 5.5 g with a mean of 1.9 g. These accessions with a 100-seed weight in the range from 2.5 to 5.0 g were 14.6% of the total. There were three accessions with 100-seed weight heavier than 5.0 g. The protein content was relatively high, ranging from 39.9% to 49.3% with a mean of 45.4%. The percentage of protein content was mostly in the range of 45.0% and 48.0%, which accounted for 55.2% of the total accessions. In contrast, the oil content was pretty low and ranged from 7.6% to 14.2%, with a mean of 10.0%. The accessions with an oil content higher than 11.5% accounted for 8.3% of the total. The correlation coefficient between the 100-seed weight and the protein content was 0.16, whereas the correlation coefficient between the 100-seed weight and the oil content was 0.29. It is interesting to notice that the seed size and protein/oil content might be correlated for wild soybean.

### 3.2. Genetic Variation

We have genotyped a total of 96 wild soybean accessions using 50 simple sequence repeat (SSR) markers in this study. These SSR markers were evenly distributed into the well-established soybean linkage groups [13]. All SSR markers provided unambiguous bands and presented a total of 198 alleles across all accessions, with an average of 3.96 alleles per locus (Table 1). The mean of major allele frequency was about 0.425. There were 14 loci (28%) with the allele number greater than 5. The least polymorphic markers (two SSR markers) and the most polymorphic markers (three SSR markers) amplified two and six alleles, respectively.

The polymorphism information content (PIC) value is an indication of allelic diversity and frequency among the individuals analyzed [14]. The PIC values ranged from 0.319 to 0.759 for the 50 SSR markers in this study, with an average of 0.619 and the majority between 0.5 and 0.7 (Table 1). The lowest PIC value was identified in the SSR marker satt130, and the highest PIC value was identified in the SSR marker satt556. About a third of the SSR markers in this study were noteworthy considering both PIC value and allele number. This is perhaps due to both the simultaneously higher PIC value and the relatively higher polymorphism. Therefore, these gene loci might need further analysis between wild soybeans and cultivated soybeans.

### 3.3. Clustering Analysis

**Figure 2:**
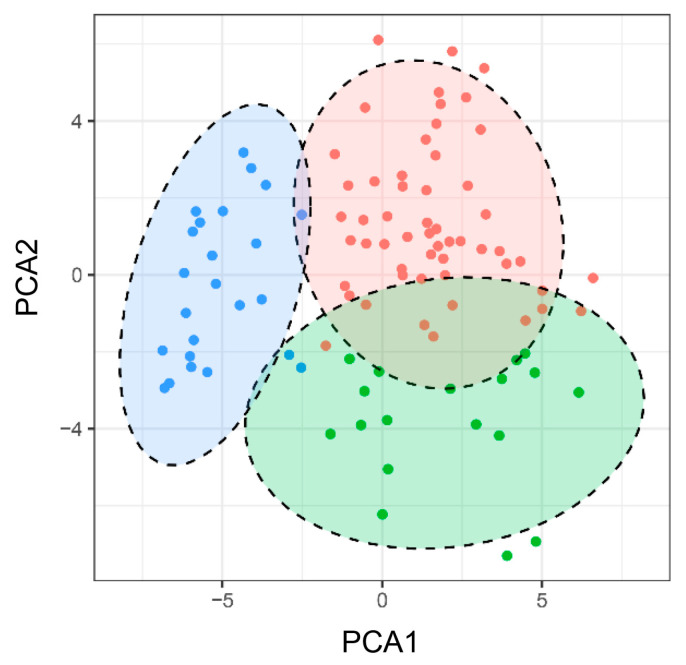
Principal component analysis of the wild soybean based on the SSR markers. Each dot represents an individual accession. Dots with the same color belong to one sub-population.

**Figure 3:**
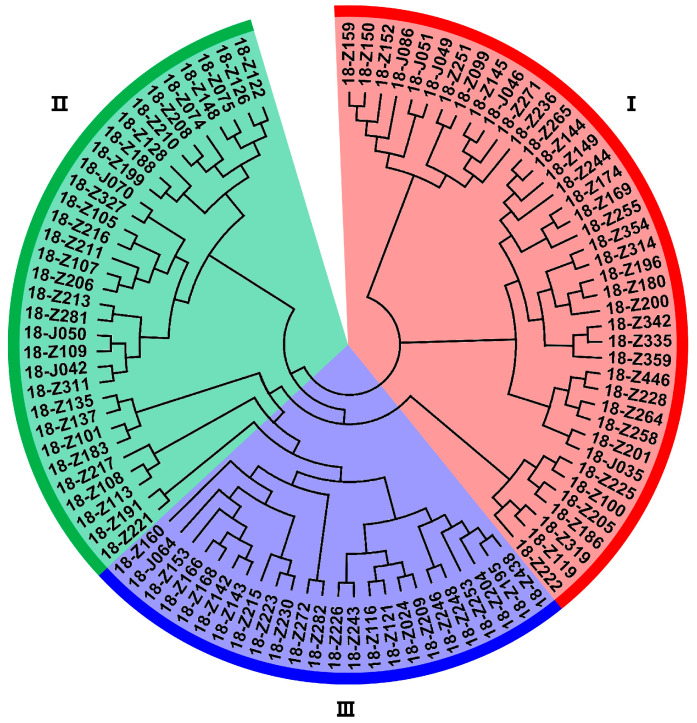
Clustering of the wild soybean based on the SSR markers. Accessions with the same color belong to one group.

The principal component analysis (PCA) exhibited the relatively close relationships between the accessions according to the genotyping data of 50 SSR markers. The majority of wild soybean accessions were distributed into three subpopulations, except for several outliers (Figure 2). Combined with the PCA result, these wild soybean accessions could be classified into three groups according to the unweighted pair group method using arithmetic mean (UPGMA) cluster analysis. Groups I, II and III contained 40, 32 and 24 accessions, respectively (Figure 3).

**Figure 4:**
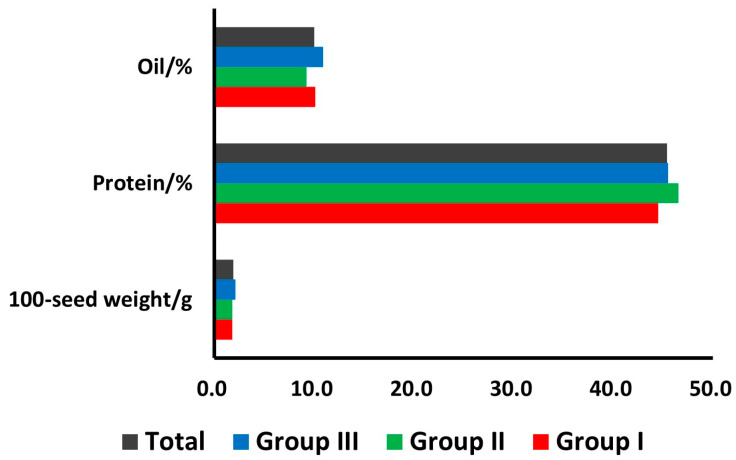
Means of seed oil and protein contents and 100-seed weight of the total wild soybean as well as three different clustering groups.

The UPGMA clustering showed a consistency with both the geographical sources and the seed quality traits. It is notable that most of these materials have been collected from the mountain areas. The materials of Group I were mainly collected from the northwest of Zhejiang, including Linan, Fuyang, Anji and Deqing Counties. The materials of Group II were mostly collected from the Western and Southern regions of Zhejiang, including Kaihua, Changshan, Suichang and Songyang Counties. However, the materials in Group III showed a geographical difference, including not only some resources collected from Tiantai and Fenghua Counties in the Eastern Zhejiang, but also some resources collected from Pingyang County in the Southern Zhejiang. The means of 100-seed weight, protein content and oil content were calculated within each group (Figure 4). The means of 100-seed weight for Groups I, II and III were 1.8, 1.8 and 2.1 g, respectively. The means of the protein content for each group were 44.5%, 46.8% and 45.5%, respectively. The means of the oil content for each group were 10.1%, 9.2% and 10.9%, respectively. The mean values of all these three traits in Group I were greater than those of the total. It is also interesting to point out that most of the accessions from Group II showed the high protein content but various agronomic performances.

## 4. Discussion

The wild soybean is a self-pollinated crop that spreads very widely in a warm and moist environment around the Northeast, the Yellow River Valley and the Southeast Coast of China [15]. Zhejiang Province is located in the southeastern coastal area of China, on the southern wing of the Yangtze River Delta. There is a long history of soybean cultivation in this province with a rich and diverse variety of soybean germplasm resources that have been formed by both natural and artificial selection through a long-term growing process [16]. Accompanied by significant changes in the earth’s ecological climate, land management methods and soil cultivation systems, especially the impact of rapid industrialization and urbanization in recent years, a large number of wild soybean resources have rapidly decreased due to the destruction of their habitats for survival and reproduction [17]. However, the quantity of commercial varieties grown has become increasingly limited with the application and promotion of current soybean breeding lines. Consequently, most landraces and wild species are in great danger of extinction now, and the reduction of germplasm resources is relatively obvious in Southeastern China [6]. The breeding parents are often limited to only a few soybean varieties with good comprehensive traits, leading to an extremely low utilization of the germplasm resources for breeding new varieties.

The genetic diversity is often analyzed using SSR markers as an effective and informative tool [13,18,19]. There were a total of 50 SSR markers applied for genotyping in this study. The average number of alleles per loci was 3.96, whereas the average PIC value was 0.619. These two parameters in this study were comparable to those in some of the previous studies [2,4,15,20,21,22]. There was an obvious genetic differentiation among the three major regions (the Northeast, the Yellow River Valley and the Yangtze River Valley) in China. However, the wild soybean in the Yangtze River valley possessed a lower genetic variation compared with those in Northeast China. We also found 19 SSR markers amplified less than 10 alleles in this study. There were significant variations in frequency among the loci in the same chromosome (linkage group). These differences might declare that the level of polymorphism for SSR loci was relatively various and depended on the genetic populations analyzed. Therefore, some of the SSR markers might need to be adjusted to be more specific for further wild soybean genotyping.

Both the phenotyping and genotyping results suggested that wild soybean resources were relatively diverse, which were also comparable to the phenomena with the landraces (traditional cultivars) in Zhejiang [6,10,11]. The clustering of most individuals had obvious regional characteristics, although a few individuals with geographically distant sources were also clustered within the same group. For example, Group I included several materials collected from Lishui and Group III included some materials collected from Linan and Fuyang. These indicated that wild soybeans in different regions might have mutual transmission and exchange under the influence of external factors. However, we’ve noticed that this event in wild soybeans is not as common as cultivated soybeans. Furthermore, the intraspecies gene recombination event is extremely important for the ecological adaptability and genetic diversity evolution of wild soybean.

A relatively narrowed genetic background is progressively generated by an excessive usage of limited elite lines owned by each breeder, which will be contrary to the soybean industry [23,24]. The genetic basis of commercial varieties has gradually been narrowed, and it is difficult to achieve a breakthrough in either yield or important quality traits [7]. This study gave us a visualized understanding of the genetic diversity and phenotypic variation of wild soybean planted in Zhejiang. Wild soybean resources in China have a number of outstanding characteristics, such as high protein content, wide adaptability and strong stress resistance. While the protein content of cultivated soybeans is about 40% [6,10], the average protein content of wild soybeans was 45.4%, with the highest reaching 49.3% in this study. Furthermore, wild soybean has developed a strong environmental adaptability during long-term evolution, including strong tolerance to abiotic stresses such as salt, aluminum ion, drought and poor soil [25,26,27]. They also exhibit good resistance to major pests and diseases that affect both quality and yield, such as mosaic virus, cyst nematode and anthracnose [28,29,30]. Reasonable crossing of germplasms between wild and cultivated soybeans is highly recommended from different clusters or with outliers based upon this result. Moreover, it is also highly encouraged to make both regional and national exchanges of elite soybean germplasms between breeders.

## 5. Conclusions

There is a long history of soybean cultivation with different types and rich resources in Zhejiang Province. However, the genetic background of current commercial varieties is relatively limited. Hence, wild soybean could provide various genetic resources for further breeding utilization in the locality. In this study, a total of 96 wild soybean accessions have been collected and then evaluated for two consecutive years. The comprehensive analyses showed agronomic and quality variations in these wild accessions. Most of the accessions exhibited sooty seed coats with different stem and leaf shapes. The protein content of half the accessions was higher than 45.5%. There were significant differences in both seed protein content and seed oil content according to the seed size. Meanwhile, the 96 individuals were genetically analyzed using 50 SSR markers, and the average number of alleles per loci was 3.96 with an average PIC value of 0.619. These accessions were then clustered into three different groups, and each group had a consistency with both the geographical sources and the seed quality traits. This study provided us a clear understanding of both phenotypic variation and genetic diversity of wild soybean in Zhejiang. Therefore, it is highly recommended to apply reasonable exchanging and crossing of different soybean types by introducing wild soybean genetic resources.

## Figures and Tables

**Table 1:** SSR locus, linkage group with position, chromosome number, of alleles found, gene diversity, heterozygosity and polymorphism information content (PIC) of all markers.

Primer Name	Linkage Group	Position (cM)	Chromosome Number	No. of Alleles	Gene Diversity	Heterozygosity	PIC
satt300	A1	30.93	5	5	0.255	0.790	0.947
satt236	A1	80.81	5	3	0.538	0.600	0.272
satt429	A2	162.02	8	4	0.375	0.715	0.238
satt197	B1	49.07	11	4	0.691	0.489	0.245
satt453	B1	123.95	11	4	0.447	0.690	0.947
satt577	B2	6.05	14	5	0.269	0.783	0.650
satt168	B2	46.87	14	5	0.353	0.731	0.147
satt556	B2	63.25	14	6	0.326	0.789	0.926
satt565	C1	5.74	4	3	0.423	0.644	0.321
satt194	C1	13.37	4	2	0.663	0.447	0.410
satt281	C2	38.90	6	4	0.494	0.651	0.517
satt371	C2	145.47	6	4	0.432	0.689	0.874
satt184	D1a	16.13	1	5	0.408	0.743	0.293
satt267	D1a	57.34	1	3	0.467	0.639	0.189
satt005	D1b	83.41	2	5	0.296	0.768	0.806
satt216	D1b	n/a	2	3	0.413	0.647	0.500
satt002	D2	42.69	17	5	0.429	0.722	0.152
satt226	D2	85.15	17	5	0.342	0.759	0.400
satt386	D2	125.00	17	3	0.483	0.607	0.144
sat-112	E	8.67	15	4	0.382	0.716	0.892
satt268	E	44.27	15	4	0.407	0.713	0.231
satt230	E	71.30	15	3	0.634	0.529	0.268
satt586	F	33.70	13	5	0.324	0.732	0.920
satt146	F	37.14	13	4	0.330	0.736	0.585
satt334	F	51.20	13	4	0.390	0.723	0.588
sct-188	F	85.33	13	4	0.452	0.589	0.938
satt130	G	23.10	18	2	0.726	0.398	0.159
satt352	G	50.52	18	3	0.482	0.616	0.500
satt442	H	43.39	12	4	0.326	0.736	0.809
satt434	H	105.73	12	3	0.542	0.589	0.281
satt571	I	14.97	20	3	0.489	0.603	0.301
satt239	I	29.61	20	5	0.389	0.750	0.522
sct_189	I	109.27	20	5	0.484	0.666	1.000
satt414	J	37.04	16	3	0.410	0.658	0.506
satt596	J	39.63	16	3	0.474	0.628	0.295
satt431	J	82.03	16	4	0.375	0.708	0.938
satt242	K	14.73	9	3	0.440	0.647	0.560
satt588	K	93.44	9	4	0.399	0.720	0.819
sat-099	L	69.12	19	4	0.339	0.711	0.736
satt373	L	93.95	19	4	0.397	0.695	0.598
satt590	M	7.75	7	3	0.356	0.666	0.200
satt346	M	106.12	7	4	0.393	0.714	0.663
satt530	N	32.84	3	4	0.300	0.746	0.756
satt387	N	53.25	3	3	0.581	0.571	0.353
satt339	N	60.17	3	6	0.323	0.765	0.948
satt022	N	84.45	3	4	0.402	0.713	0.141
satt487	O	9.53	10	4	0.349	0.731	0.914
satt173	O	58.40	10	5	0.259	0.792	0.805
satt345	O	59.43	10	3	0.617	0.533	0.255
satt243	O	107.31	10	6	0.354	0.763	0.831

## Data Availability

The raw data supporting the conclusions of this article will be made available by the authors on request.
